# Suspected Cardioembolic Stroke Linked to Left Atrial Stunning After Pulsed Field Ablation for Atrial Fibrillation

**DOI:** 10.1002/joa3.70327

**Published:** 2026-05-04

**Authors:** Akinori Matsushima, Takamichi Katsuhara, Tomonobu Kodama, Kenta Uto, Yasuo Okumura

**Affiliations:** ^1^ Division of Cardiology, Department of Medicine Nihon University School of Medicine Tokyo Japan; ^2^ Division of Neurosurgery, Department of Neurological Surgery Nihon University School of Medicine Tokyo Japan; ^3^ Division of Human Pathology, Department of Pathology and Microbiology Nihon University School of Medicine Tokyo Japan

**Keywords:** atrial fibrillation, pulsed field ablation, stroke

## Abstract

After pulsed field ablation with pulmonary vein and posterior wall isolation, a patient developed ischemic stroke due to left carotid artery occlusion. Pathology of the retrieved thrombus revealed an acute red thrombus, suggesting left atrial stunning–induced thrombus formation.

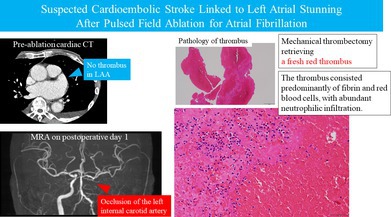

Left atrial (LA) stunning is well recognized after cardioversion for atrial fibrillation (AF) and has recently been reported following pulsed field ablation (PFA). We report a case suggesting that cerebral infarction may have resulted from thrombus formation due to LA stunning.

A 78‐year‐old man under surveillance for a left internal iliac artery aneurysm was incidentally found to have bilateral pleural effusions on CT and reported 2 months of exertional dyspnea. ECG revealed new‐onset AF, and laboratory tests showed elevated NT‐proBNP (10 361 pg/mL). He was admitted with acute heart failure presenting with dyspnea at rest, orthopnea, and peripheral edema. Transthoracic echocardiography showed a left ventricular ejection fraction (LVEF) of 31.6% with diffuse hypokinesis, LA volume 121.5 mL (LAVI 73.2 mL/m^2^), mild aortic regurgitation, and moderate mitral regurgitation. His CHADS_2_ score was 3 (heart failure, age ≥ 75 years, and hypertension), CHA_2_DS_2_‐VASc score was 4 (heart failure, age ≥ 75 years, and hypertension), and HAS‐BLED score was 1 (age > 65 years).

Initial therapy included diuretics, β‐blocker, digoxin, edoxaban, and aprindine. Sinus rhythm was restored by cardioversion after transesophageal echocardiography (TEE) confirmed no thrombus but revealed spontaneous echo contrast with a left atrial appendage (LAA) emptying velocity of 28.8 cm/s. He was later readmitted with recurrent heart failure and, after stabilization, underwent pulmonary vein isolation and posterior LA wall isolation using the FARAPULSE PFA system. Preablation cardiac CT again excluded thrombus (Figure 1). Edoxaban 30 mg, which the patient had been receiving preprocedurally, was uninterrupted throughout the peri‐ and postprocedural periods. Immediately after transseptal puncture, 7000 units of heparin were administered intravenously, and an activated clotting time (ACT) exceeding 400 s was confirmed 9 min later, after which pulmonary vein isolation was initiated. Throughout the procedure, all ACT measurements remained above 350 s, with the final ACT recorded at 352 s. Sinus rhythm was restored spontaneously after pulmonary vein isolation. After withdrawal of all catheters and sheaths from the left atrium, 40 mg of protamine was administered to reverse the effect of heparin, and the procedure was uneventful. Edoxaban 30 mg was resumed on the day following the procedure. On postoperative day 1 at 18:07, however, he developed dysarthria and altered consciousness. Emergency MRI performed at 19:05 revealed multiple microinfarctions (Figure 2A), and MRA showed left internal carotid artery occlusion (Figure 2B). Emergency cerebral angiography was subsequently performed at 20:12. At that time, the left internal carotid artery lesion was considered to represent chronic severe stenosis, and given a National Institutes of Health Stroke Scale (NIHSS) score of 2 with no progression of neurological symptoms, a conservative management strategy was initially adopted. On the following day, neurological symptoms persisted with progression to right‐sided incomplete hemiparesis, prompting repeat brain magnetic resonance imaging. Diffusion‐weighted imaging demonstrated an increase in scattered cerebral infarcts, raising suspicion of an acute occlusive process. Consequently, emergency cerebral angiography was repeated at 10:48 on postoperative day 2. At 11:30, mechanical thrombectomy achieved reperfusion (Figure 2C), retrieving a fresh red thrombus. The reperfusion grade was Thrombolysis in Cerebral Infarction (TICI) grade 3. Immediately after the procedure, improvement was observed in the level of consciousness, dysarthria, and right‐sided upper and lower extremity weakness. Pathology confirmed acute cardioembolism (Figure 2D). Neurological symptoms resolved, and he was discharged without deficits. The modified Rankin Scale score at discharge was 0.

**FIGURE 1:**
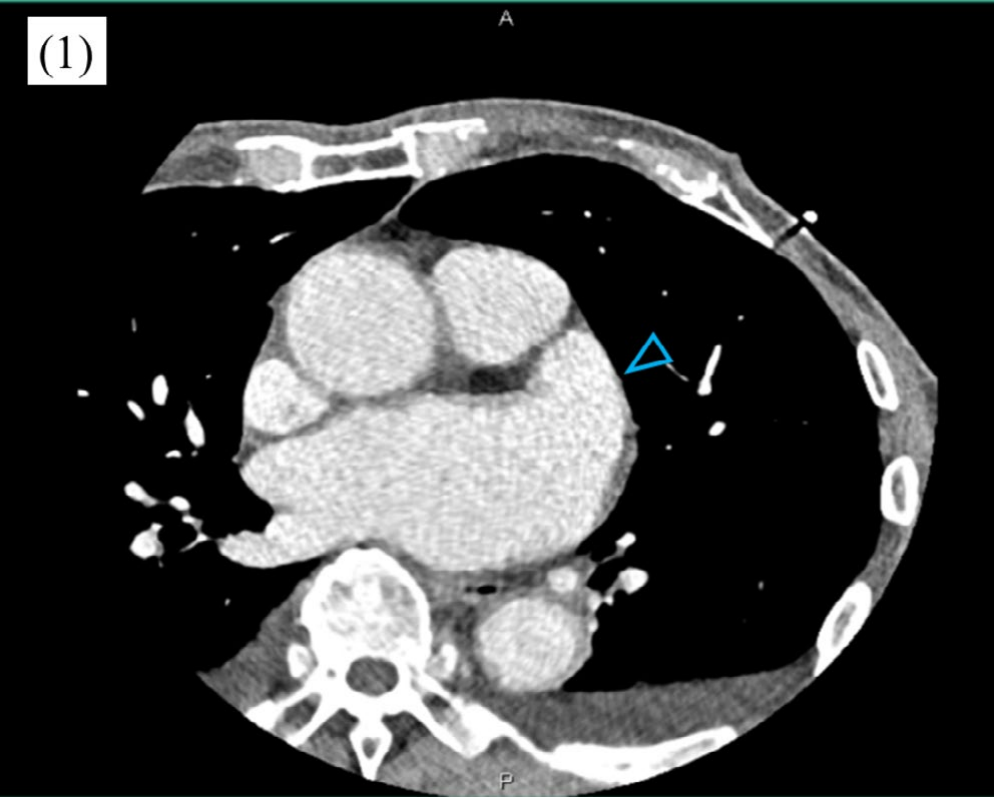
Contrast‐enhanced cardiac computed tomography prior to ablation showing no filling defects in the left atrium (LA) or left atrial appendage (LAA). The LAA is indicated by the blue arrow.

**FIGURE 2:**
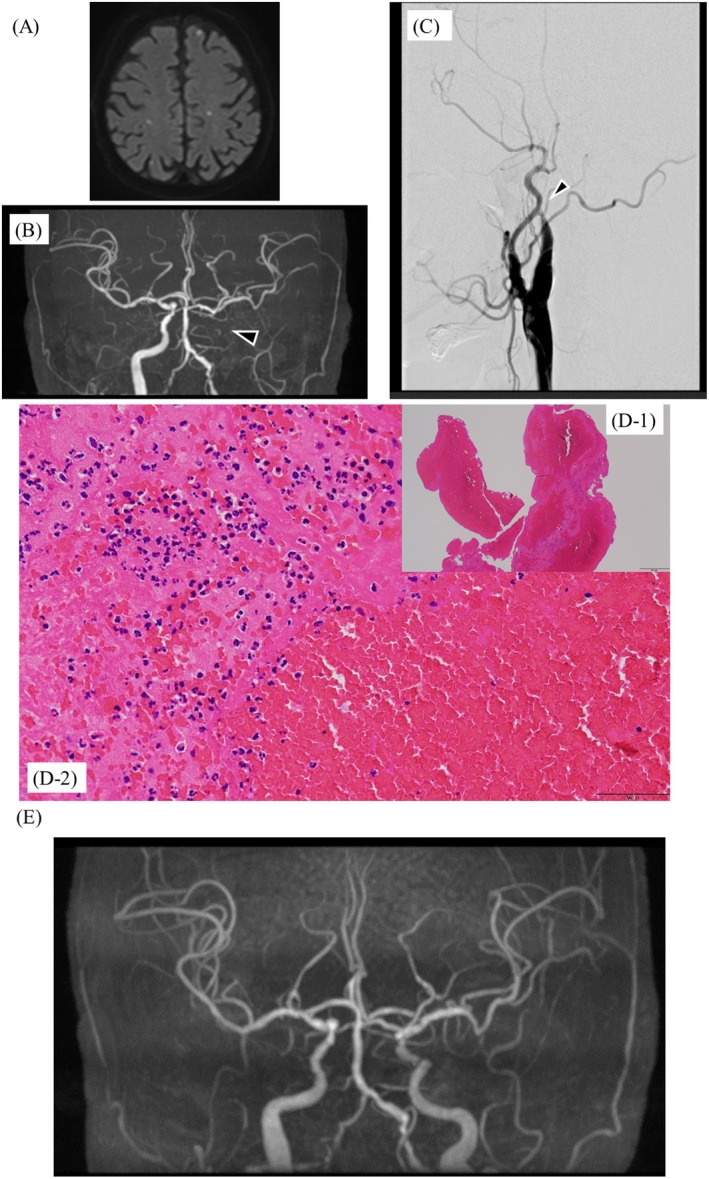
Diffusion‐weighted brain MRI (A), MRA (B), and cerebral angiography (C) obtained on the day following ablation; hematoxylin and eosin staining at low magnification (D‐1) and high magnification (D‐2); and brain MRA performed 8 days after mechanical thrombectomy of the left internal carotid artery (E). The arrowheads indicate occlusion of the left internal carotid artery.

This patient developed acute ischemic stroke one day after PFA for AF, possibly related to ablation‐induced LA stunning with de novo thrombus formation. LA stunning is well documented after cardioversion [1] and has also been observed after PFA [2, 3]. Lakkireddy et al. (NEMESIS‐PFA trial) demonstrated impaired LA contractile function immediately after PFA without structural damage [2], while Verhaeghe et al. reported greater LA strain reduction after PFA compared with radiofrequency ablation [3], indicating an energy‐specific stunning effect. Although direct postprocedural assessment of LA function was not available in this case, the absence of preprocedural thrombus and spontaneous restoration of sinus rhythm during PFA support the hypothesis that transient postablation atrial dysfunction may have played a contributory role.

In addition, the procedure occurred during recovery from acute heart failure. Acute heart failure and elevated NT‐proBNP levels are known to transiently enhance thrombotic propensity through systemic inflammatory responses, endothelial dysfunction, and activation of coagulation pathways [4]. Furthermore, this patient had marked LA enlargement (left atrial volume index 73.2 mL/m^2^), indicating a pre‐existing structural substrate prone to blood stasis, which may have synergistically increased the risk of thrombus formation in the peri‐procedural period.

An additional noteworthy aspect of this case is the clinical course of the stroke itself. Because neurological symptoms were initially mild and the left internal carotid artery lesion was considered to represent chronic severe stenosis, a conservative strategy was initially chosen. However, symptom progression prompted reassessment, and delayed mechanical thrombectomy successfully achieved complete reperfusion (TICI grade 3), resulting in full neurological recovery (mRS 0). Pathological analysis of the retrieved thrombus confirmed an acutely formed cardioembolic clot, supporting the diagnosis of an acute embolic event rather than progression of chronic cerebrovascular disease.

Previous work has shown that in AF patients with a history of ischemic stroke undergoing catheter ablation, recurrence of cardioembolic stroke is rare, suggesting that not all prior strokes in this population are attributable to AF [5]. In contrast, our patient developed an acute cardioembolic event shortly after ablation, highlighting that ablation‐related stunning and the peri‐procedural prothrombotic milieu can still pose a risk even when preprocedural imaging excludes thrombus and anticoagulation is uninterrupted.

In summary, this case suggests that PFA may induce transient LA stunning and, in susceptible patients, promote acute thrombus formation, leading to embolic stroke, particularly in patients recovering from acute heart failure with severe LA enlargement. Careful patient selection, optimal timing after stabilization, strict anticoagulation, and vigilant postoperative monitoring are crucial to minimize complications. Early recognition and timely mechanical thrombectomy may enable complete neurological recovery, even when initial symptoms are mild.

## Funding

The authors have nothing to report.

## Ethics Statement

The authors have nothing to report.

## Consent

Informed consents were obtained from the patients to publish the spotlight.

## Conflicts of Interest

Dr. Okumura received research grants unrelated to this study from Johnson & Johnson KK and Biosense Webster Inc., scholarship funds from Nippon Boehringer Ingelheim, and remuneration from Daiichi‐Sankyo, AstraZeneca, Bayer Healthcare, Bristol‐Myers Squibb, and Johnson & Johnson KK. Also, he belongs to the endowed departments of Boston Scientific Japan, Biotronik Japan, Abbott Medical Japan, Japan Lifeline, and Medtronic Japan. Other authors have no conflicts of interest for this article.

## Data Availability

The data that support the findings of this study are available on request from the corresponding author. The data are not publicly available due to privacy or ethical restrictions.

## References

[1] I. A. Khan , “Transient Atrial Mechanical Dysfunction (Stunning) After Cardioversion of Atrial Fibrillation and Flutter,” American Heart Journal 144 (2002): 11–22, 10.1067/mhj.2002.123113. 12094183

[2] Y. Nakatani , S. Sridi‐Cheniti , G. Cheniti , et al., “Pulsed Field Ablation Prevents Chronic Atrial Fibrotic Changes and Restrictive Mechanics After Catheter Ablation for Atrial Fibrillation,” Europace 23 (2021): 1767–1776, 10.1093/europace/euab155. 34240134 PMC8576285

[3] D. Lakkireddy , A. Katapadi , J. Garg , et al., “NEMESIS‐PFA: Investigating Collateral Tissue Injury Associated With Pulsed Field Ablation,” JACC: Clinical Electrophysiology 11 (2025): 1747–1756, 10.1016/j.jacep.2025.04.017. 40392666

[4] L. Verhaeghe , W. L'Hoyes , J. Vijgen , et al., “Left Atrial Function After Atrial Fibrillation Ablation With Pulsed Field Versus Radiofrequency Energy: A Comparative Study,” Heart Rhythm (2025), 10.1016/j.hrthm.2025.07.043. 40744192

[5] S. Fukamizu , R. Hojo , T. Kitamura , et al., “Recurrent Ischemic Stroke in Patients With Atrial Fibrillation Ablation and Prior Stroke: A Study Based on Etiological Classification,” Journal of Arrhythmia 36 (2020): 95–104, 10.1002/joa3.12285. 32071627 PMC7011801

